# Body size–trophic position relationships among fishes of the lower Mekong basin

**DOI:** 10.1098/rsos.160645

**Published:** 2017-01-04

**Authors:** Chouly Ou, Carmen G. Montaña, Kirk O. Winemiller

**Affiliations:** 1 Department of Wildlife and Fisheries Sciences, Texas A&M University, 2258 TAMU, College Station, TX 77843, USA; 2 Department of Biological Sciences, Sam Houston State University, 1900 Avenue I, Huntsville, TX 77341, USA

**Keywords:** benthic, Cambodia, food web, floodplain river, guild, pelagic

## Abstract

Body size is frequently claimed to be a major determinant of animal trophic interactions, yet few studies have explored relationships between body size and trophic interactions in rivers, especially within the tropics. We examined relationships between body size and trophic position (TP) within fish assemblages in four lowland rivers of the Lower Mekong Basin in Cambodia. Stable isotope analysis (based on δ^15^N) was used to estimate TP of common fish species in each river, and species were classified according to occupation of benthic versus pelagic habitats and major feeding guilds. Regression analysis yielded strong correlations between body size and TP among fishes from the Sesan and Sreprok rivers, but not those from the Mekong and Sekong rivers. The Mekong fish assemblage had higher average TP compared with those of other rivers. The relationship between body size and TP was positive and significantly correlated for piscivores and omnivores, but not for detritivores and insectivores. The body size–TP relationship did not differ between pelagic and benthic fishes. Body size significantly predicted TP within the orders Siluriformes and Perciformes, but not for Cypriniformes, the most species-rich and ecologically diverse order in the Lower Mekong River. We conclude that for species-rich, tropical fish assemblages with many detritivores and invertivores, body size would not be an appropriate surrogate for TP in food web models and other ecological applications.

## 1. Background

Body size is recognized as an important determinant of community structure because it influences ecological processes that affect consumer–resource interactions, life-history traits, population dynamics and metabolic rates [1–6]. Body size has been incorporated in food web models that seek to predict ecosystem stability, patterns of energy flow and response to disturbances [7,8]. Food webs are frequently structured by body size, such that predators are larger than their prey and larger individuals feed at higher trophic levels [4,9].

The relationship between animal body size and trophic position (TP) has long been a major topic of discussion among ecologists, and a hierarchy of increasing body size with increasing trophic level has been broadly accepted since Charles Elton's work in the early 1900s [4,10]. TP might increase with body size because larger prey satisfy the higher metabolic demands of larger consumers more efficiently [11] or because consumer mouth gape constrains the upper limit of prey sizes that can be ingested [12,13], or both. Empirical studies in marine and freshwater ecosystems have supported the positive relationships between body size and TP. For example, analysis of a global dataset of 8361 fish species from 57 orders found a significant positive relationship between body size and TP [14]. Similar findings are reported by Nakazawa *et al*. [15], who investigated relationship between body size and δ^15^N (an index of relative TP) using long-term data (40 years) for freshwater fishes from Lake Biwa, Japan. Although there is ample evidence of positive relationships between body size and TP of fishes, other studies have shown no relationship between TP and size [16–18] or a negative relationship [19,20], which suggests that phylogeny and associated morphological and behavioural constraints also influence how body size affects species interactions [20,21]. Thus, even though the body size–TP relationship in fishes appears to be strongly influenced by gape limitation [13,22], other morphological traits could play roles [14].

Given the mixed findings for the relationships between TP and body size, there is a need for further empirical investigation and refinement of theories. Although many studies have revealed a significant relationship between body size and trophic level in fishes, most research has been conducted in temperate marine and freshwater ecosystems, and only one investigation appears to have been conducted in tropical freshwater [18]. Tropical rivers support the highest freshwater fish diversity, and many have high productivity that supports important fisheries. Therefore, a better understanding of the relationship between body size and TP in these ecosystems has immediate resource management applications [23,24].

Here, we used data from fish specimens collected from four tropical rivers of the Lower Mekong River Basin in Southeast Asia to examine the relationship between body size and TP. Specifically, we tested for relationships between TP and body size at the taxonomic level (order), guild (habitat and trophic) level and assemblage level across four rivers. Given the great ecological diversification apparent among tropical freshwater fishes, we predicted the body size–TP relationship in tributaries of the Lower Mekong would be influenced by multiple traits rather than only gape limitation. Better understanding of body size–TP relationships in local assemblages is critical for numerous ecological applications that are based on community size spectra [23].

## 2. Material and methods

### 2.1. The Mekong river basin and study sites

The Mekong is one of the world's major rivers in terms of size, productivity, biodiversity, economic impact and cultural significance [25,26]. The Mekong River ranks third in fish species diversity after the Amazon in South America and the Congo in Africa [27]. Over 1000 fish species, belonging to 24 orders and 87 families, and more than 200 endemic freshwater species have been documented in the Mekong Basin [28]. Fisheries production from the Mekong is estimated at 2 million tons per year [29], which is more than 20% of the World's inland capture [30]. Approximately 75 million people living in Southeast Asia, particularly those who live in the Lower Mekong Basin, depend on inland fisheries for food security. More than 70% of protein intake among Cambodians is reported to derive from wild-caught fish [31]. Pressure on Lower Mekong fisheries has intensified in recent years due to rapid economic and population growth. Many fishers in the region, particularly in Cambodia, have reported declines in catches of large catfishes (e.g. pangasids) and overall reductions in fish size over the past 30 years [31]. Such changes are consistent with the ‘fishing-down-the-food-web’ model in which progressively smaller and less valuable species are exploited as larger and more valuable stocks are depleted [7,32].

We sampled fish assemblages in four rivers in northeastern Cambodia: the Mekong, Sekong, Sesan and Srepok rivers. The latter three rivers are major tributaries of the Lower Mekong and often are referred to as the 3S rivers (see Ou & Winemiller [33] for more details about these rivers). The stretch of the Mekong River and its riparian zone between Stung Treng and the Laos–Cambodia border was designated a RAMSAR wetland of global significance because of its biodiversity conservation value in the Indo-Burma region. Watersheds of the 3S rivers also have been identified as critical areas for biodiversity conservation [34].

### 2.2. Sample collections and analysis

We collected samples of fish tissues and important production sources for stable isotope analysis from one reach (approx. 3 km) in each of the four rivers during the dry season (January 2010 and January 2011). Production sources included benthic filamentous algae, phytomicrobenthos (benthic microscopic algae and associated biofilm), seston (phytoplankton and other suspended fine particulate matter) and common riparian plants. Whenever possible, tissue samples from 3–5 adult specimens of each common fish species and 3–5 samples of production sources were retained from each site for analysis. Different plant parts (leaves, fruits and seeds) were collected, cut into small pieces, placed in plastic bags and preserved in salt for later analysis in the laboratory. Phytomicrobenthos samples were collected by scraping rocks and submerged tree branches. Seston samples were collected from near the water surface with 1-l opaque bottles, and the water was filtered through pre-combusted Whatman GF/F filters (pore size 0.7 µm).

Fishes were collected using multiple gears including seines, cast nets and dip nets. Additional fish specimens were obtained from local fishers who primarily fished with gill nets and baited hooks. Fish specimens were identified to species level and measured to the nearest 1.0 mm standard length (SL). Fish muscle tissue samples were taken from the flank near the base of the dorsal fin. Fish tissue samples were preserved in salt and processed following methods of Arrington & Winemiller [31]. In the laboratory, tissue samples were soaked in distilled water for 4–5 h, rinsed and dried in an oven at 60°C for 48 h [35]. Dried samples were ground into fine powder using an electronic ball-mill grinder then stored in clean glass vials. Subsamples were weighed to the nearest 0.02 mg and packaged into Ultra-Pure tin capsules. Samples were analysed for stable isotope ratios of nitrogen (^15^N/^14^N) at the Analytical Chemistry Laboratory of the Institute of Ecology at the University of Georgia. Results of isotope ratios are reported in parts per thousand (‰) compared with standard values of atmospheric nitrogen as δX = [(*R*_sample_/*R*_standard_) − 1]× 10^3^, where *R* = ^15^N/^14^N.

### 2.3. Trophic position calculation

Stable isotopes of nitrogen have been widely used for estimating TP of metazoans in food web studies [36]. For each fish species, TP was calculated using the formula [36,37]: TP = (δ^15^N_consumers _−δ^15^N_basal source_)/2.5 + 1, where δ^15^N_consumers_ is δ^15^N signature of fishes, and δ^15^N_basal source_ is the mean δ^15^N value of primary production sources: phytomicrobenthos, seston and leaves of riparian macrophytes. The value 2.5 represents trophic fractionation of the isotopic ratio (the shift that occurs in material between its ingestion by a consumer and its assimilation into the consumer's tissue); here, we used the mean trophic fractionation value derived from a meta-analysis of laboratory feeding studies involving diverse metazoan consumers [38]. When calculating TP of fish specimens or species, the mean δ^15^N of primary production sources was based on samples obtained from the same survey locality where the fishes were collected (estimates of production sources supporting fishes at each site presented in Ou & Winemiller [33]).

### 2.4. Classification of species trophic and habitat guilds

Fishes were classified into four trophic guilds and two habitat guilds according to information obtained from FishBase [39] and Rainboth [40], as well as interpretation of fish functional morphology (electronic supplementary material, 1). The four trophic guilds are: (i) *piscivore*: fish that consume mostly fish and, in some cases, lesser proportions of decapod crustaceans or other macroinvertebrates; (ii) *omnivore:* trophic generalists that consume variable proportions of phytoplankton, benthic algae and aquatic or terrestrial invertebrates; (iii) *detritivore:* fish that consume detritus and/or algae; and (iv) *insectivore:* fish with diets strongly dominated by insects or other invertebrates such as aquatic microcrustaceans. The two habitat guilds are (i) *benthic*: fishes adapted to live in the benthic zone or bottom of the water column, (ii) *pelagic*: fishes that live in the open water column away from the bottom.

### 2.5. Statistical analysis

We used linear regression to explore relationships between body size and TP. Analyses of the body size–TP relationship were performed using 699 fish specimens belonging to 143 species, 73 genera, 26 families and 7 orders (electronic supplementary material, 1). Our surveys were designed to capture most of the common species and some of the uncommon species within a given river reach. As multiple gear types and habitats were surveyed within each reach, samples were assumed to reflect size structures of local populations of the common species at those locations during the dry season. All specimens were adult size classes, which should minimize influence from allometry. Body size and TP were log-transformed before performing analyses. Linear regression analyses were performed separately on three taxonomic orders that contained more than 10 species (Cypriniformes, Perciformes, Siluriformes), four trophic guilds and two habitat guilds. One-way analysis of variance (ANOVA) was used to test whether distributions of body size and TP differed between guilds and rivers. Analyses were performed using the software PAST [41]. Because a molecular time-calibrated phylogeny with branch lengths is not available for this diverse fish assemblage, we created a phylogenetic distance matrix of fish species using taxonomic classification levels to explore the influence of phylogeny on the relationship between body size and TP [42]. This proxy method provides a crude but reasonable estimate of evolutionary relationships among species within diverse assemblages. We estimated phylogenetic distances based on the taxonomy presented by Rainboth *et al*. [28]. A Mantel test was used to examine relationships between shared phylogenetic dissimilarity estimated from taxonomic distance, body size and TP, and a partial Mantel test was used to evaluate the influence of phylogenetic distance on the relationship between body size and TP [21].

## 3. Results

**Figure 1.**
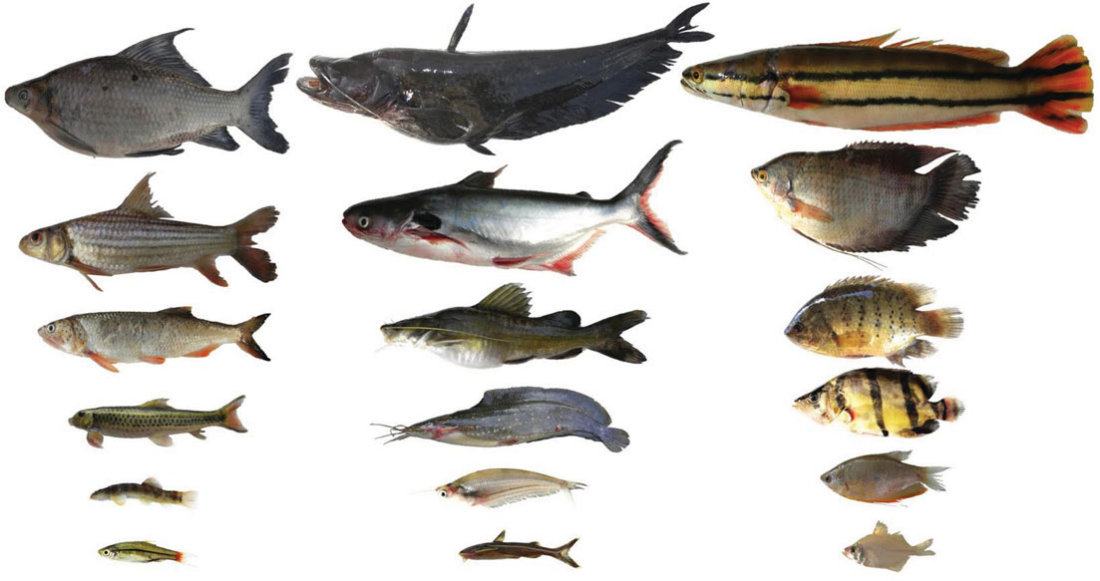
Examples of a range of body sizes among fish species of the lower Mekong River Basin: Cypriniformes (left column, species listed from top to bottom, values are standard lengths in centimetres)—*Labeo chrysophekadion* 36, *Probarbus jullieni* 23, *Raimas guttatus* 21, *Garra fasciacauda* 12.5, *Schistura* sp. 4, *Rasbora borapetensis* 3.5; Siluriformes (middle column)—*Wallago micropogon* 65, *Pangasius larnaudii* 53, *Hemibagrus filamentus* 21, *Clarias melanoderma* 19, *Kryptopterus bicirrhus* 9.5, *Glyptothorax laosensis* 6; Perciformes (right column)—*Channa micropeltes* 60, *Osphronemus gouramy* 25, *Pristolepis fasciata* 16.5, *Datnioides undecemradiatus* 13, *Trichopodus trichopterus* 8.5, *Parambasis siamensis* 4.5.

Across all four fish assemblages, the order Cypriniformes was the most abundant in samples, followed by Siluriformes and Perciformes (electronic supplementary material, 1). The most abundant family was Cyprinidae followed by Siluridae and Bagridae (electronic supplementary material, 1). The orders Cypriniformes, Siluriformes and Perciformes were represented across all trophic and habitat guilds. When both guilds are considered simultaneously, benthic detritivores were represented by cypriniforms only. A wide range of fish body lengths was obtained across all sites (figure 1), including loaches as small as 3 cm (*Schistura* sp., Nemacheilidae) to snakeheads (*Channa micropeltes*, Channidae) measuring up to 60 cm SL. There were no significant relationships between phylogenetic distance and TP (Mantel test, *r* = 0.04, *p* = 0.8), phylogenetic distance and body size (*r* = −0.02, *p* = 0.80), or phylogenetic distance and the relationship between body size and TP (Partial Mantel test, *r* = −0.02, *p* = 0.82).

**Figure 2.**
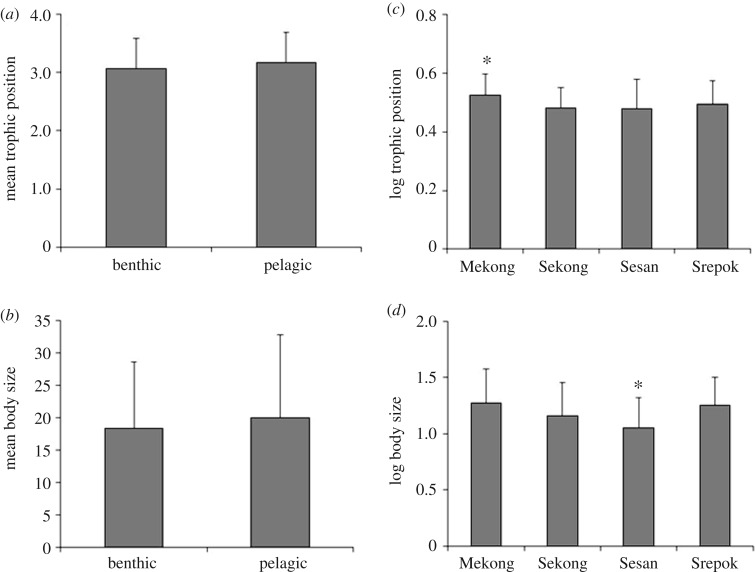
Mean (±s.d.) of trophic position (*a*) and body size (*b*) of fish assemblages in benthic versus pelagic habitats, and of trophic position (*c*) and body size (*d*) across four rivers. Asterisk above bar denotes significant difference (*p* < 0.05) from other groups.

**Figure 3.**
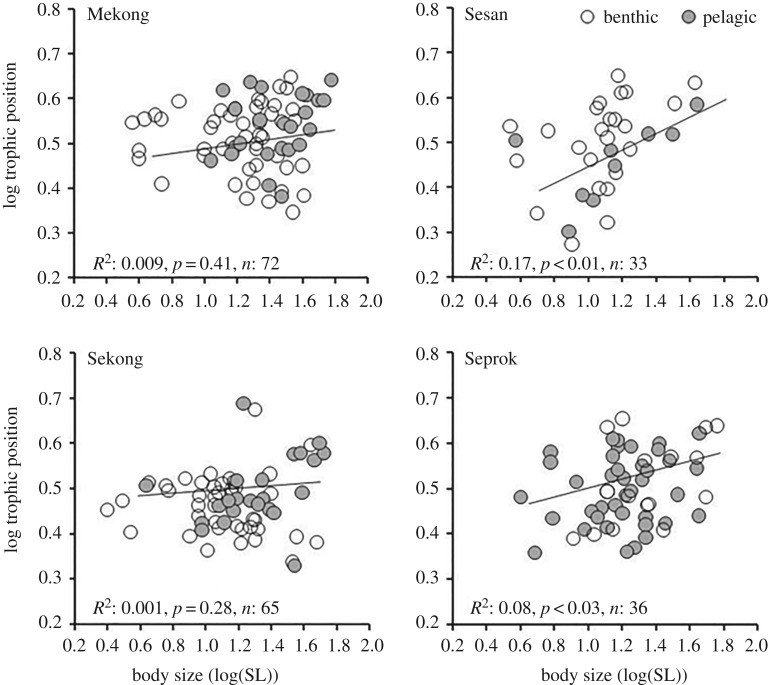
Relationship between body size and trophic position of fish assemblages in benthic and pelagic habitats across all four rivers.

Distributions of body size and TP did not differ significantly between benthic and pelagic fishes (figure 2*a*,*b*). Among trophic guilds, detritivores had the lowest mean TP (2.6) followed by omnivores (2.8), insectivores (3.3) and piscivores (3.6) (electronic supplementary material, 1). TP varied significantly among fish assemblages of the four rivers (*F*_(1,486)_ = 7.72, *p* < 0.001; figure 2*c*), with Mekong fishes having higher TP on average (Tukey HSD, *p* < 0.001). Body size also differed among rivers (*F*_(1,486)_ = 14.02, *p *< 0.001; figure 2*d*), and average body size of Sesan fishes was significantly smaller (Tukey HSD, *p *< 0.01) (electronic supplementary material, S2). Regression analysis indicated significant correlations between body size and TP for fishes inhabiting the Sesan and Seprok rivers, but not in the Mekong and Sekong rivers (figure 3).

**Figure 4.**
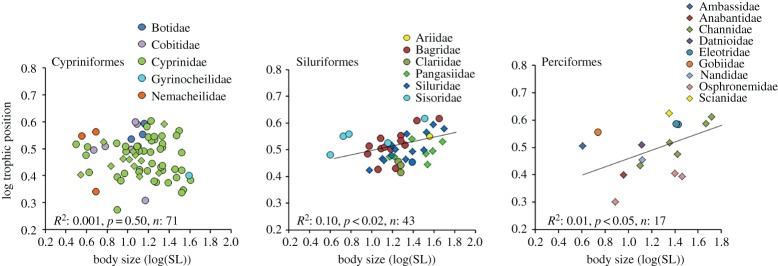
Body size–TP relationships of fish assemblages among three taxonomic orders. Families within each taxonomic order are colour coded. Circles represent benthic species, diamonds represent pelagic species.

**Figure 5.**
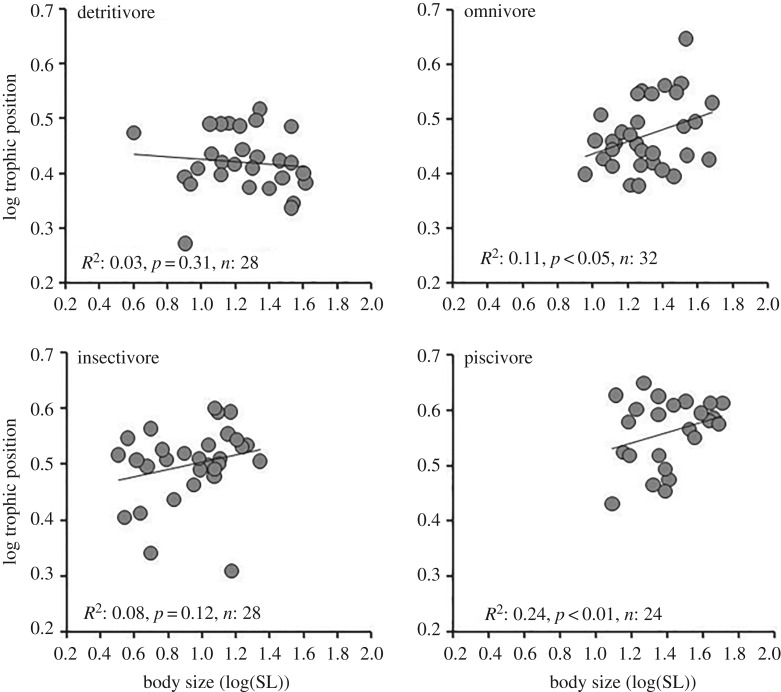
Body size–TP relationships of fish assemblages among four trophic guilds.

A significant positive relationship was obtained between body size and TP for Siluriformes and Perciformes, but body size did not predict higher TP in Cypriniformes (figure 4), and this regression slope actually was negative. The relationship between fish body size and TP was not significant for detritivores or insectivores, but a statistically significant relationship was obtained for piscivores and omnivores (figure 5).

## 4. Discussion

Fishes of the Lower Mekong Basin are extremely diverse in terms of body size, habitat use and feeding ecology. This diversity has been attributed to many factors, including the complex historical biogeography of the region [40], seasonal flood pulsing and its effects on habitat and food web dynamics [43], and greater niche diversification in the tropics compared with temperate regions [44]. Perhaps not surprisingly, our study identified contrasting patterns for the body size–TP relationship among taxa, local species assemblages and habitat guilds.

Overall, our findings did not support the hypothesis that mouth gape constrains prey ingestion and tropic interactions to produce a strong, positive body size–TP relationship, as proposed elsewhere[10–12,14,22]. In biodiverse tropical food webs, mouth gape does not seem to constrain feeding efficiency of detritivorous and insectivorous fishes. For instance, both small (e.g. *Henicorhynchus siamensis*, 4 cm SL) and large-bodied fishes (e.g. *Incisilabeo behri*, 41 cm SL) in the Lower Mekong River are detritivores. For piscivores that ingest individual prey whole, gape limitation would be expected to produce a positive body size–TP relationship. However, the substantial range of prey body sizes in topical food rivers, including many fishes that are primary consumers, might not translate into a strong positive relationship because piscivores of all sizes can exploit suitably sized prey that span a wide range of trophic positions [18].

Cypriniformes, a species-rich order that dominates freshwater systems in Asia and North America [45], has many detritivorous, algivorous and omnivorous species in the Lower Mekong that span a wide range of adult body sizes, including members of pelagic and benthic habitat guilds. Perciformes and Siluriformes also have species in both habitat guilds and represent a broad range of trophic groups, with insectivores, omnivores and piscivores common. The body size–TP relationship was positive and significant for Mekong Perciformes and Siluriformes, groups dominated by species that ingest relatively large food items and therefore may often be gape limited, and was weaker and non-significant for Mekong Cypriniformes, a group that contains many microphagous detritivores and insectivores that span a wide range of body sizes (figure 1) [40]. The regression slope for cypriniforms was negative, a finding consistent with studies of cypriniforms from other regions [14,20]. In addition to large cypriniforms that feed at low trophic positions, the lack of correlation between body size and TP in the cypriniform assemblage dataset (figure 4) also is influenced by the presence of small species with relatively high TPs. For example, small loaches (e.g. Cobitidae, Nemacheilidae) that feed on benthic invertebrates sometimes have relatively high trophic positions (3.1–4) whereas other small cypriniforms have low TPs. Our body size–TP regression for siluriforms also was influenced by some large catfishes with relatively low trophic positions (e.g. *Clarias macrocephalus*, TP 2.6) and small catfishes with intermediate to high trophic positions (e.g. *Glyptothorax laoensis*, TP 3.6). Interestingly, one of the largest freshwater fishes in the world, the Mekong giant catfish (*Pangasianodon gigas*, 3 m), is described as an omnivore [40].

The lack of relationship between body size and TP in cyprinids has been attributed to their evolutionary history [20,46,47]. German *et al*. [47] suggested that certain morphological/physiological traits of cyprinids have allowed them to overcome constraints that generally yield a positive body size–TP relationship. Cyprinids possess well-developed pharyngeal teeth used for processing food prior to digestion [46], and many have long digestive tracts that increase gut passage time to increase efficiency of plant material digestion. These two anatomical modifications appear to have played significant roles in the evolution of dietary specialization within the family [46,47]. Most of the largest cyprinids in the Lower Mekong are detritivores or omnivores that consequently have low trophic positions, and smaller cyprinids encompass a broad range of feeding habits and trophic positions. One can only speculate why large predatory cyprinids are not more common in the Mekong. The largest piscivorous cyprinid in our local assemblages was *Macrochirichthys macrochirus* (mean SL 22.5 cm, mean TP 3.9); two larger piscivorous cyprinids (*Aaptosyax grypus* maximum SL 130 cm, *Luciocyprinus striolatus* max. SL 200 cm) occur in the middle Mekong (Laos, Thailand), where they now are rare.

The body size–TP relationship did not differ between benthic and pelagic fishes, probably because both groups exploit similarly broad ranges of food resources. Our study did, however, reveal differences in the body size–TP relationship among trophic guilds. The positive relationships observed for piscivores and omnivores could be explained by optimal foraging theory and/or mouth gape constraint. For example, piscivores in the Lower Mekong River probably attack the most abundant and largest prey among those encountered and capable of being ingested. The most commonly encountered forage fishes probably are detritivores and omnivores. Loaches and other small benthic insectivores tend to be less abundant and more cryptic. In the Lower Mekong, some large piscivorous fishes indeed are positioned relatively low in the food web, indicating that they feed heavily on detritivores and omnivores at low trophic levels. Food chains supporting top predators in tropical rivers tend to be short (3–4 levels) [48]. For example, during subsidence of the annual flood pulse in a Venezuelan river, large piscivores (*Cichla temensis*) fed heavily on a large detritivore (*Semaprochilodus kneri*) that occupies a low TP [18]. When consumers exploit abundant prey at lower trophic levels, these short food chains should increase ecological efficiency [49,50]. An analysis of gut contents of several piscivorous fishes from Venezuelan rivers indicated that predator mouth gape, prey size and prey abundance all influenced size distributions of consumed prey [51]. However, there are cases in which piscivore size and prey size are uncorrelated, for example species that feed on scales, fins or mucus of other fishes [52,53]. None of the piscivorous fishes in our dataset have been reported to exhibit these feeding strategies [40].

Absence of a body size–TP relationship among detritivorous fishes has been reported in several other studies [17,20,54], perhaps because detritivorous fishes of any size can consume fine particulate organic matter. In tropical rivers, detritivorous and algivorous fishes are particularly diverse in terms of body size, morphology and habitat use [55]. The guild of detritivorous/algivorous fishes in the Lower Mekong spans a broad range of body sizes. For example, there is a 10-fold difference in the body size of the detritivorous/algivorous cyprinids *Garra fasciacauda* and *Labeo chrysophekadion*. Body size did not appear to predict TP among insectivores, and this similarly could be explained by a lack of mouth gape limitation for this guild; both small and large fishes can feed on small aquatic invertebrates. In the Lower Mekong, body length of benthic insectivorous fishes ranged from 2 cm (e.g. *Schistura* sp.) to approximately 90 cm (e.g. *Mastacembelus armatus*).

Significant body size–TP relationships were obtained only for fish assemblages of the Sesan and Srepok rivers, and not the Sekong and Mekong mainstem. Compared with the other rivers, the Sesan has relatively few large piscivores, yet some relatively small species, such as *Yasuhikotakia modesta*, had relatively high TPs (i.e. more than 4). Overall, our sample from the Sesan had lower fish richness and fewer large migratory species (electronic supplementary material, S2). The Sesan's hydrology is regulated by upstream dams, with the loss of high flow pulses that increase longitudinal and lateral habitat connectivity and sediment dynamics that maintain deep pool habitats in the channel; this in turn has affected the Sesan fish assemblage and food web ecology [33]. The Srepok River, by contrast, has been much less impacted by dams and has relatively high fish diversity (approx. 240 species) [56]. The Sreprok has more deep pools than the Sesan and supports more large migratory fishes, including several with TP > 4. The Mekong and Sekong surveys yielded samples with greater species richness and wider ranges of body sizes and feeding habits compared with those from the Sesan and Srepok. Predators in more species-rich and complex food webs can exploit prey from wider spectra of body sizes and trophic positions that include large primary consumers, thus weakening the body size–TP relationship [18].

The body size–TP relationship of Lower Mekong fishes differed among the three major taxonomic orders, but there also exists phylogenetic, body size and trophic variation within each order that was not formally analysed given the lack of a phylogeny for all taxa. Use of taxonomic distance as a proxy for phylogenetic distance in the Mantel tests did not reveal a significant relationship with body size. Neither body size nor TP distributions differed significantly among the three major taxonomic orders (electronic supplementary material, S2). For example, Cypriniformes in this dataset ranged in size from 3 cm (e.g. *Schistura* sp.) to 41 cm SL (*I. behri*), and Siluriformes also have very small (*Glyptothorax lampris*, 4 cm) and large species (*Hemibagrus wyckioides*, 45 cm). The most species-rich families within these orders (e.g. Cyprinidae, Siluridae, Bagridae) had broadly overlapping distributions for both body size and TP distributions (figure 4). Some closely related species had divergent trophic positions that might reflect adaptative divergence [57]. For example, *Channa gachua* and *Ch. micropeltes*, two snakeheads, had different body sizes (12.6 versus 51.8 cm SL, respectively) and trophic levels (2.7 versus 4.1, respectively). Costa [58] also used taxonomy as a proxy for phylogeny of marine predators, and found no effect of phylogeny on the body size–TP relationship. By contrast, recent study by Naisbit *et al*. [21], in which taxonomy was used to represent phylogeny, found that closely related species had similar trophic levels. Based on analysis of molecular phylogenetic information for sharks, Rezende *et al*. [59] found that phylogeny significantly influenced the body size–TP relationship. Without further analysis using robust phylogenetic data, it is not possible to determine if phylogenetic relationships explain some of the variation in our datasets.

Our findings largely contradict the broadly accepted assumption of the *fishing-down-the-food-web* model. Pauly *et al*. [7] proposed that fish body size is positively correlated with TP, so that removal of the most valuable and largest fish from a system results in a reduction in average food chain length. This model was largely based on commercial fisheries in marine pelagic systems, and might not be applicable for the Lower Mekong and other river-floodplain ecosystems that have high taxonomic and ecological diversity. Tropical river-floodplain ecosystems support important subsistent fisheries that are critical for regional food security, especially for the rural poor. Fishers in this region preferentially target high-value species, which generally are the largest. In tropical inland fisheries, these species can have trophic positions that are either low (detritivores, herbivores) or high (piscivores, insectivores). Body size is not a useful surrogate for vertical TP in tropical freshwater systems containing diverse detritivores and insectivores, but body size can be useful for predicting feeding relationships among piscivores, especially those that ingest prey whole and are constrained by mouth gape. Caution is warranted in the application of body size–TP relationships to assess threats to freshwater biodiversity. We contend that the *fishing-down-the-food-web model* is not applicable to rivers of the Lower Mekong. This is not to say that there has not been a *fishing-down of body size*. Overharvest of large fishes already has resulted in greater reliance on harvest of small fishes that have high demographic resilience and diverse trophic positions [56,60] with unknown consequences for fishery productivity [61].

A better understanding of the relationship between body size and TP in these ecosystems has immediate resource management applications. For instance, ontogenetic shifts toward higher trophic positions are generally assumed based on the larger absolute size of mouth gape of larger conspecifics. In some cases, TP declines as fishes grow (e.g. herbivorous and detritivorous fishes). When fisheries remove large herbivorous and detritivorous fishes from these food webs, diets of predatory fishes may shift and consequently affect secondary production [18]. A strong relationship between body size and TP is often assumed in food web and ecosystem models of stock or biomass dynamics used to inform fisheries management (e.g. [62]). Likewise, a strong relationship is generally assumed when estimating bioaccumulation of toxins, such as heavy metals and pesticides, in fish tissues. Our findings indicate that, at least in the case of biodiverse fish assemblages of tropical rivers, this assumption does not hold, and it probably requires further study for verification in other systems.

## Supplementary Material

Supplementary material 1

## Supplementary Material

Supplementary material 2

## Data accessibility

Our data are deposited as the electronic supplementary material at http://rsos.royalsocietypublishing.org.
