# Supplementary material for: Body size–trophic position relationships among fishes of the lower Mekong basin
Source: R Soc Open Sci. 2017 Jan 4;4(1):160645. doi: 10.1098/rsos.160645 (PMC5319329; doi:10.1098/rsos.160645)
Supplement: Supplementary material 1 [file rsos160645supp1.pdf]

Supplementary material 1. Taxonomy, trophic positions and body sizes of fishes from the Lower Mekong River Basin (River abbreviations are M (Mekong), Sk (Sekong), Sr (Srepok) and Ss (Sesan).

Order	Family	Species name	Habitat	Trophic guild	Mean standard length (SL)	Mean trophic position (TP)	Rivers
Beloniformes	Belonidae	*Xenentodon cancila*	P	P	18.1	3.5	M, Sk, Sr
Cypriniformes	Botiidae	*Syncrossus helodes*	B	I	14.8	3.9	M, Ss
Cypriniformes	Botiidae	*Yasuhikotakia caudipunctata*	B	I	11	3.4	M,
Cypriniformes	Botiidae	*Yasuhikotakia modesta*	B	I	14.3	3.6	M, Sk, Ss
Cypriniformes	Cobitidae	*Acantopsis* cf. *gracilentus*	B	I	15	2	Sr
Cypriniformes	Cobitidae	*Acantopsis* sp. 1	B	I	12.5	3.9	M, Sk, Ss
Cypriniformes	Cobitidae	*Acantopsis* sp. 2	B	I	12	4	M, Sk
Cypriniformes	Cobitidae	*Acantopsis* sp. 3	B	I	11	3.1	Sk, Sk
Cypriniformes	Cobitidae	*Acantopsis* sp. 4	B	I	4.8	3.1	Sk,Ss
Cypriniformes	Cobitidae	*Acantopsis* sp. 5	B	I	6.2	3.2	Sr
Cypriniformes	Cyprinidae	*Barbonymus altus*	B	O	13.5	2.9	M, Sk, Sr
Cypriniformes	Cyprinidae	*Barbonymus gonionotus*	B	O	22	2.6	Sr
Cypriniformes	Cyprinidae	*Barbonymus schwanenfeldii*	B	O	10.3	2.9	Ss
Cypriniformes	Cyprinidae	*Cirrhinus jullieni*	B	D	8	2.5	Sk
Cypriniformes	Cyprinidae	*Cirrhinus microlepis*	B	D	35	2.2	M
Cypriniformes	Cyprinidae	*Cosmocheilus harmandi*	B	O	32	3.7	M, Sr
Cypriniformes	Cyprinidae	*Cyclocheilichthys apogon*	B	I	9.8	3.1	Ss
Cypriniformes	Cyprinidae	*Cyclocheilichthys enoplus*	B	I	22	3.2	M
Cypriniformes	Cyprinidae	*Cyclocheilichthys furcatus*	B	I	18.5	3.4	Sk
Cypriniformes	Cyprinidae	*Cyclocheilichthys lagleri*	B	I	11.8	3	M
Cypriniformes	Cyprinidae	*Cyclocheilichthys mekongensis*	B	I	12.8	3.2	Sk, Ss, Sr

Cypriniformes	Cyprinidae	*Cyclocheilichthys tapiensis*	B	I	17.3	3.4	M
Cypriniformes	Cyprinidae	*Garra fasciacauda*	B	D	11.5	2.7	Sr
Cypriniformes	Cyprinidae	*Henicorhynchus lobatus*	B	D	8.7	2.4	M, Sk
Cypriniformes	Cyprinidae	*Henicorhynchus siamensis*	B	D	4	3	M
Cypriniformes	Cyprinidae	*Hypsibarbus lagleri*	B	D	29	2.6	Sr
Cypriniformes	Cyprinidae	*Hypsibarbus malcolmi*	B	D	22	3.3	M
Cypriniformes	Cyprinidae	*Hypsibarbus pierrei*	B	D	34	3.1	Sr
Cypriniformes	Cyprinidae	*Hypsibarbus* sp.	B	D	8	1.9	Ss
Cypriniformes	Cyprinidae	*Hypsibarbus wetmorei*	B	D	30	2.5	M
Cypriniformes	Cyprinidae	*Incisilabeo behri*	B	D	41	2.4	M
Cypriniformes	Cyprinidae	*Labeo chrysophekadion*	B	D	34	2.6	M, Sk
Cypriniformes	Cyprinidae	*Labiobarbus leptocheilus*	B	D	13.3	2.6	Sk, Ss, Sr
Cypriniformes	Cyprinidae	*Labiobarbus siamensis*	B	D	17.5	2.8	M, Sr
Cypriniformes	Cyprinidae	*Leptobarbus hoevenii*	B	D	34	2.2	Sk
Cypriniformes	Cyprinidae	*Lobocheilos rhabdoura*	B	D	14.5	3.1	Sk
Cypriniformes	Cyprinidae	*Lobocheilos melanotaenia*	B	D	16.8	3.1	Sr
Cypriniformes	Cyprinidae	*Mekongina erythrospila*	B	D	25	2.4	M
Cypriniformes	Cyprinidae	*Osteochilus vittatus*	B	D	20	2.6	M
Cypriniformes	Cyprinidae	*Osteochilus lini*	B	D	9.5	2.6	Sr
Cypriniformes	Cyprinidae	*Osteochilus melanopleurus*	B	D	21	3.1	M
Cypriniformes	Cyprinidae	*Osteochilus schlegelii*	B	D	13	3.1	M, Ss
Cypriniformes	Cyprinidae	*Osteochilus waandersii*	B	D	15.7	2.6	Sk
Cypriniformes	Cyprinidae	*Oxygaster anomalura*	P	I	14	3.3	Sk
Cypriniformes	Cyprinidae	*Probarbus jullieni*	B	O	21.8	3.5	M
Cypriniformes	Cyprinidae	*Probarbus labeaminor*	B	O	22	2.7	Sr
Cypriniformes	Cyprinidae	*Puntioplites bulu*	B	O	13	2.6	Sr
Cypriniformes	Cyprinidae	*Puntioplites falcifer*	B	O	12.4	3.2	M, Sk, Sr
Cypriniformes	Cyprinidae	*Scaphognathops bandanensis*	B	O	18.3	2.4	M
Cypriniformes	Cyprinidae	*Scaphognathops stejnegeri*	B	O	18.2	3.5	M, Sr

Cypriniformes	Cyprinidae	*Tor sinensis*	B	O	30	3.5	M
Cypriniformes	Gyrinocheilidae	*Gyrinocheilus pennocki*	B	D	40	2.8	M
Cypriniformes	Nemacheilidae	*Nemacheilus longistriatus*	B	I	5	3.7	M
Cypriniformes	Nemacheilidae	*Schistura* sp. 1	B	I	3.7	3.5	M, Ss
Cypriniformes	Cyprinidae	*Hampala dispar*	P	P	17.1	4	M, Ss,, Sr
Cypriniformes	Cyprinidae	*Hampala macrolepidota*	P	P	15.6	3.8	Sk, Ss, Sr
Cypriniformes	Cyprinidae	*Macrochirichthys macrochirus*	P	P	22.5	3.9	M
Cypriniformes	Cyprinidae	*Mystacoleucos marginatus*	P	I	9.6	3.2	M, Sr
Cypriniformes	Cyprinidae	*Opsarius pulchellus*	P	I	4.1	3.2	M, Sk
Cypriniformes	Cyprinidae	*Parachela siamensis*	P	I	9	2.9	Sk
Cypriniformes	Cyprinidae	*Paralaubuca typus*	P	I	7.9	3.3	M, Sk
Cypriniformes	Cyprinidae	*Poropuntius laoensis*	P	I	12	3.1	Sk
Cypriniformes	Cyprinidae	*Poropuntius normani*	P	D	19.3	2.4	Sk, Sr
Cypriniformes	Cyprinidae	*Puntius orphoides*	P	D	13	2.5	Ss
Cypriniformes	Cyprinidae	*Puntius* sp.	P	D	11.3	3.1	Ss, Sr
Cypriniformes	Cyprinidae	*Raiamus guttatus*	P	I	16.2	3.5	M, Ss
Cypriniformes	Cyprinidae	*Rasbora* sp. 1	P	I	5.8	3.4	Sk, Ss
Cypriniformes	Cyprinidae	*Rasbora paviana*	P	I	4.3	2.6	Ss, Sr
Cypriniformes	Cyprinidae	*Rasbora* sp. 2	P	I	3.2	3.3	Ss
Cypriniformes	Cyprinidae	*Rasbora tornieri*	P	I	6.9	2.7	Sk, Sr
Cypriniformes	Cyprinidae	*Rasbora trilineata*	P	I	3.5	2.5	Sk
Osteoglossiformes	Notopteridae	*Notopterus notopterus*	P	I	18.7	3	M, Sk, Ss, Sr
Osteoglossiformes	Notopteridae	*Chitala blanci*	P	P	43.5	3.8	M, Sr
Perciformes	Gobiidae	*Papuligobius ocellatus*	B	I	5.5	3.6	M
Perciformes	Ambassidae	*Parambassis siamensis*	P	I	4.1	3.2	Sk, Ss
Perciformes	Anabantidae	*Anabas testudineus*	P	O	9.1	2.5	M, Sk, Ss, Sr
Perciformes	Channidae	*Channa gachua*	P	P	12.6	2.7	Ss, Sr
Perciformes	Channidae	*Channa lucius*	P	P	22.7	3.3	Sk, Ss
Perciformes	Channidae	*Channa marulioides*	P	P	46.3	3.9	M, Sr

Perciformes	Channidae	*Channa micropeltes*	P	P	51.8	4.1	M, Sk, Sr
Perciformes	Channidae	*Channa striata*	P	P	26.5	3	M, Sk, Ss, Sr
Perciformes	Datnioiddiae	*Datnioides undecimradiatus*	P	P	13	3.2	M, Sr
Perciformes	Eleotridae	*Oxyeleotris marmorata*	P	I	27	3.8	M, Sk, Sr
Perciformes	Nandidae	*Pristolepis fasciata*	P	O	13.2	2.8	M, Ss, Sr
Perciformes	Osphronemidae	*Trichopodus trichopterus*	P	I	7.8	2	Ss, Sr
Perciformes	Osphronemidae	*Osphronemus exodon*	P	O	29	2.5	M
Perciformes	Osphronemidae	*Osphronemus goramy*	P	O	25	2.5	M
Perciformes	Sciaenidae	*Boesemania microlepis*	P	P	22.5	4.2	M
Pleuronectiformes	Soleidae	*Brachirus harmandi*	B	I	26	3.7	M
Pleuronectiformes	Cynoglossidae	*Cynoglossus feldmanni*	B	I	28	3	M
Siluriformes	Ariidae	*Hemiarius verrucosus*	B	P	36	3.5	M
Siluriformes	Bagridae	*Bagrichthys majusculus*	B	O	17.1	2.7	Sk, Sr
Siluriformes	Bagridae	*Bagrichthys obscurus*	B	O	19	3.6	Sk
Siluriformes	Bagridae	*Hemibagrus filamentus*	B	I	21	3.3	Sr
Siluriformes	Bagridae	*Hemibagrus nemurus*	B	I	19.1	3.4	Sr
Siluriformes	Bagridae	*Hemibagrus spilopterus*	B	I	12.3	3.2	M, Ss
Siluriformes	Bagridae	*Hemibagrus wyckii*	B	P	27.3	4.1	M, Ss, Sr,
Siluriformes	Bagridae	*Hemibagrus wyckioides*	B	P	44.9	4.1	M, Sk, Ss, Sr
Siluriformes	Bagridae	*Mystus albolineatus*	B	I	9.5	3.3	Sk
Siluriformes	Bagridae	*Mystus bocourti*	B	I	15.3	3.2	Sk
Siluriformes	Bagridae	*Mystus multiradiatus*	B	I	9.2	3	Sk
Siluriformes	Bagridae	*Mystus singaringan*	B	I	12.2	3.5	M, Ss, Sr
Siluriformes	Bagridae	*Pseudomystus siamensis*	B	I	13.3	3.2	Sr
Siluriformes	Clariidae	*Clarias batrachus*	B	O	17.9	2.9	M, Sk, Ss,
Siluriformes	Clariidae	*Clarias macrocephalus*	B	O	19	2.6	Sk
Siluriformes	Clariidae	*Clarias melanoderma*	B	O	19	2.8	M
Siluriformes	Sisoridae	*Glyptothorax fuscus*	B	I	5.3	3.5	Sr
Siluriformes	Sisoridae	*Glyptothorax lampris*	B	I	4	3	Sr

Siluriformes	Sisoridae	*Glyptothorax laoensis*	B	I	6	3.6	Sr
Siluriformes	Sisoridae	*Bagarius bagarius*	B	P	14.3	3.3	Sk, Sr
Siluriformes	Sisoridae	*Bagarius suchus*	B	P	32.3	4.1	M, Sk
Siluriformes	Pangasiidae	*Helicophagus waandersii*	B	O	24.4	3.5	M, Sk, Sr
Siluriformes	Pangasiidae	*Pangasianodon hypophthalmus*	P	O	33	3.1	M
Siluriformes	Pangasiidae	*Pangasius bocourti*	P	O	34.5	2.8	M, Sk
Siluriformes	Pangasiidae	*Pangasius conchophilus*	P	O	38.7	3.1	M, Sk
Siluriformes	Pangasiidae	*Pangasius larnaudii*	P	O	48	3.4	M, Sk, Sr
Siluriformes	Pangasiidae	*Pangasius macronema*	P	O	14.8	3	M
Siluriformes	Pangasiidae	*Pseudolais pleurotaenia*	P	O	16.4	3	Sk, Sr
Siluriformes	Siluridae	*Belodontichthys truncatus*	P	P	33.7	3.7	M, Sk
Siluriformes	Siluridae	*Hemisilurus mekongensis*	P	O	34	3.4	M
Siluriformes	Siluridae	*Kryptopterus bicirrhis*	P	I	9.5	2.6	Sk
Siluriformes	Siluridae	*Kryptopterus geminus*	P	I	15.5	3	Sk
Siluriformes	Siluridae	*Kryptopterus limpok*	P	I	12.6	2.9	Sk
Siluriformes	Siluridae	*Kryptopterus paraschilbeides*	P	P	15.5	3.3	Sk
Siluriformes	Siluridae	*Micronema cheveyi*	P	I	16.8	3.2	M,
Siluriformes	Siluridae	*Micronema moorei*	P	I	21.7	3.6	M, Sr
Siluriformes	Siluridae	*Ompok bimaculatus*	P	O	13	2.9	Sk, Sr
Siluriformes	Siluridae	*Phalacronotus apogon*	P	P	24.6	3.1	Sk, Sr
Siluriformes	Siluridae	*Phalacronotus bleekeri*	P	P	21.2	2.9	Sk
Siluriformes	Siluridae	*Wallago attu*	P	P	39.2	3.9	M, Sk
Siluriformes	Siluridae	*Wallago micropogon*	P	P	49.8	3.8	M, k
Synbranchiformes	Mastacembelidae	*Macrognathus semiocellatus*	B	I	20	2.7	Sk
Synbranchiformes	Mastacembelidae	*Macrognathus siamensis*	B	I	18.3	2.5	Sk, Ss
Synbranchiformes	Mastacembelidae	*Mastacembelus armatus*	B	I	39.2	3.7	M, Sk, Ss, Sr
Synbranchiformes	Synbranchidae	*Ophisternon bengalense*	B	O	46.5	2.7	Sk, Sr
