# Supplementary material for: Body size–trophic position relationships among fishes of the lower Mekong basin
Source: R Soc Open Sci. 2017 Jan 4;4(1):160645. doi: 10.1098/rsos.160645 (PMC5319329; doi:10.1098/rsos.160645)

Supplementary material 2. Frequency histograms of body size and trophic position based on individual fish specimens from surveys of species assemblages in four rivers in the lower Mekong River.

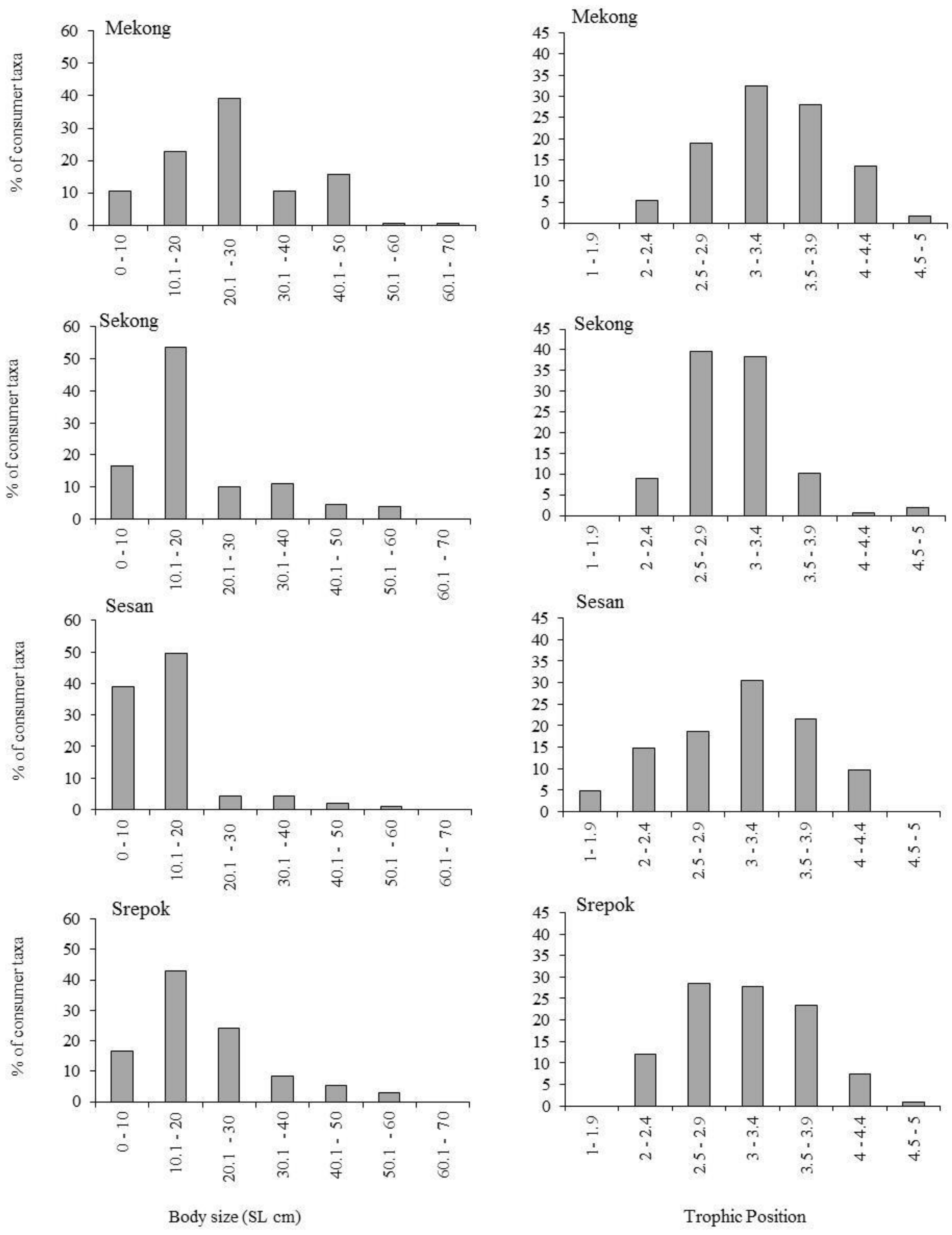

Supplement: Supplementary material 2 [file rsos160645supp2.pdf]
